# Manchester Royal Infirmary

**Published:** 1910-02-26

**Authors:** 


### MANCHESTER ROYAL INFIRMARY.

We have received the following account relating to the agitation which has been going on for months in favour of the appointment of qualified women to resident posts in this institution. It is interesting to record that, although the decision has gone against the women so far as the resident appointments are concerned, it has led to the granting of further opportunities to medical women graduates of the Victoria University to perfect themselves in the practice of their profession.

Our correspondent reports that at the close of the annual general meeting a special general meeting of trustees was held to consider a resolution submitted by Sir William Cobbett (who presided), and seconded by the Rt. Rev. Bishop Welldon, Dean of Manchester, on the subject of medical women residents. Since January, 1909, when the Board of Management received a deputation of ladies on this subject, it has been persistently attacked in the columns of the *Manchester Guardian* by a cleverly engineered series of communications assisted from time to time by editorials. The Board appointed a Special Committee, which considered the question for several months, and finally, in November last, presented a report, which negatived the claims of the ladies. The publication of this report produced a strong agitation. A committee consisting chiefly of representatives of women's societies, and of women graduates of the Victoria University of Manchester, was formed to obtain a reversal of the Board's decision, petitions were widely circulated for signature, demanding reconsideration, correspondence appeared in the *Guardian* and other local newspapers, printed matter was circulated amongst the trustees by the advocates of the ladies' claims, and a memorandum on the subject, enclosing a copy of the report of the Special Committee of the Board, was issued by the Board of Management. The resolution submitted to the meeting by the Chairman was : "That it is not desirable to appoint women to resident medical and surgical posts at the Manchester Royal Infirmary." There was a large attendance of trustees, nearly 200 being present, and after a full debate, in which, in addition to the mover and seconder, Mr. J. E. Platt, F.R.C.S. (Chairman of the Medical Board), Professors Schuster and Herford, of the Manchester University, Mr. Alfred Simpson, Dr. Reynolds, Mr. C. P. Scott (Governing Director of the *Manchester Guardian*), and other trustees took part, and a letter was read from the Vice-Chancellor of the University repudiating recognition of the agitation by the University, and declaring that the question was entirely one for the Board of Management of the Infirmary to settle, the resolution of the Board was carried by a huge majority.

The attitude taken by the Board of Management was that although they sympathised with the desire of the medical women for opportunities of gaining further experience, they considered that it would be a disadvantage to the institution and to the welfare of the patients to admit them to resident posts, that they were not such useful officers in a general hospital as male residents, and that their claim to be admitted on equal terms with men was as impracticable as it was undesirable. The speeches made in support of the decision of the Board clearly indicated that the matter had been dealt with impartially, and that although the Board could not accede to the demands so vehemently pressed upon them, they were prepared to consider more suitable methods of giving to medical women graduates of the University, who had received their practical training within the walls of the Infirmary, further opportunities of perfecting themselves in the practice of their profession.

---

The death is announced, at Cloncurry, North Queensland, of Mr. J. Campbell, at the age of thirty-four. Mr. Campbell was a Fellow of the R.C.S. of Ireland, and had been surgeon to the Cloncurry Hospital, and medical officer of health to the same locality. He had also held the posts of hon. medical officer to the N. Borneo Government and Government medical officer, Kudat, besides similar positions to various tobacco companies.

